# Alternations in the human skin, gut and vaginal microbiomes in perimenopausal or postmenopausal Vulvar lichen sclerosus

**DOI:** 10.1038/s41598-024-58983-y

**Published:** 2024-04-10

**Authors:** Xiaolei Ma, Guangdong Wen, Zheng Zhao, Lulu Lu, Tianying Li, Na Gao, Gangwen Han

**Affiliations:** 1 Department of Dermatology, Peking University International Hospital, Life Park Road No.1 Life Science Park of Zhong Guancun, Chang Ping District, Beijing, People’s Republic of China; 2 Department of Pathology, Peking University International Hospital, Beijing, People’s Republic of China; 3 Department of Dermatology, Peking University People’s Hospital, Beijing, People’s Republic of China

**Keywords:** VLS, Gut microbe, Skin microbe, Vaginal microbe, Metagenomics, KEGG database, Diseases, Skin diseases

## Abstract

Vulvar lichen sclerosus (VLS) is a chronic and progressive dermatologic condition that can cause physical dysfunction, disfigurement, and impaired quality of life. However, the etiology of VLS remains unknown. The vulvar skin, intestinal and vaginal microbiomes have been postulated to play important roles in the pathogenesis of this disease. The aim of this study was to compare the compositional characteristics of the vulvar skin, vagina, and gut microbiota between perimenopausal or postmenopausal VLS patients and healthy controls. The study involved six perimenopausal or postmenopausal VLS patients which were based on characteristic clinical manifestations and histologic confirmation and five healthy controls. The pruritus severity of each patient was evaluated using the NRS scale, and the dermatology-specific health-related quality of life was assessed using the Skindex-16. Metagenomic sequencing was performed, and the results were analyzed for alpha and beta diversity. LEfSe analysis were used to investigate the microbial alterations in vulvar skin, gut and vagina. KEGG databases were used to analyze differences in functional abundance. The study found significant differences in alpha diversity between the two groups in stool and vaginal samples (P < 0.05). Patients with VLS had a higher abundance of *Enterobacter cloacae*, *Flavobacterium_branchiophilum*, *Mediterranea_sp._An20, Parabacteroides_johnsonii*and *Streptococcus_bovimastitidis* on the vulvar skin, while *Corynebacterium_sp._zg-913* was less abundant compared to the control group. The relative abundance of *Sphingomonas_sp._SCN_67_18*, *Sphingobium_sp._Ant17,* and *Pontibacter_sp_BT213* was significantly higher in the gut samples of patients with VLS.*Paenibacillus_popilliae*,*Gemella_asaccharolytica*, and *Coriobacteriales_bacterium_DNF00809* compared to the control group. Additionally, the vaginal samples of patients with VLS exhibited a significantly lower relative abundance of *Bacteroidales_bacterium_43_8*, *Bacteroides_sp._CAG:20*, *Blautia_sp._AM28-10, Fibrobacter_sp*._*UWB16*, *Lachnospiraceae_bacterium_AM25-39, Holdemania_filiformis, Lachnospiraceae_bacterium_GAM79*, and *Tolumonas_sp*. Additionally, the *butyrate-producing bacterium SS3/4* showed a significant difference compared to the controls. The study found a negative relationship between *Sphingobium_sp._Ant17* in stool and Skindex-16 (P < 0.05), while *Mediterranea_sp._An20* had a positive correlation with Skindex-16 (P < 0.05) in the skin. Additionally, our functional analysis revealed alterations in Aminoacyl_tRNA_biosynthesis, Glutathione_metabolism, the pentose phosphate pathway, and Alanine__aspartate_and_glutamate_metabolism in the VLS patient group. The study suggests that perimenopausal or postmenopausal patients with VLS have a modified microbiome in the vulvar skin, gut, and vagina. This modification is linked to abnormal energy metabolism, increased oxidative stress, and abnormal amino acid metabolism.

## Introduction

Lichen sclerosus (LS) is a chronic dermatologic condition which accompanied by symptoms of pruritus and usually occurs in the anogenital region but can develop on any skin surface. Vulvar LS (VLS) can occur at any age but tends to have two peaks of onset: first, in prepubertal girls; second, in perimenopausal or postmenopausal women^1^ . LS is a commonly treated condition in vulvar clinics that can cause physical dysfunction, disfigurement, and impaired quality of life. The etiology of this condition remains unclear. Although several mechanisms have been proposed based on epidemiologic data, including genetic factors, immunologic abnormalities, hormonal factors, infection, cell kinetics, and local factors^2–6^, none have been definitively proven. A study using skin grafts demonstrated that a graft placed on the vulva developed LS, and skin with LS from the vulva became normal when grafted to the thigh^7^. These findings suggested that local vulvar factors may contribute to disease progression. Although infectious agents such as *Borrelia burgdorferi*, *variably acid-fast bacteria*, and *human papillomavirus*) have been suggested to induce LS^8–10^, no clear relationship has been demonstrated.

The skin is the largest organ of the body and is colonized by complex microbial communities. Abnormalities in the skin flora are often associated with various skin diseases^11,12^. A comprehensive of the skin and intestinal flora could lead to new approaches for the diagnosing and treating skin and intestinal diseases. The vagina has a unique structure that includes a microbiological system consisting of the vaginal microbiome. The normal vaginal microbiome, environment and host are interdependent and mutually restricted, maintaining a dynamic balance of the microecological system. For example, it is known to all that *lactobacilli* is a probiotic in the vagina due to its reinforcement of the host immune system against several primary and opportunistic pathogens, but its probiotic activity is caused not only by individual *Lactobacillus* species but also by its multi-microbial interaction as consortia which is influenced by numerous factors^13–15^. Disruption of any factor in the balance can lead to a breakdown of the balance, resulting in a decrease in probiotics, and potentially causing gynecological diseases^16,17^.

Few studies have focused on microbiota in perimenopausal or postmenopausal VLS. This study aimed to investigate the potential involvement of skin, gut, and vaginal microbiomes in the development of perimenopausal or postmenopausal VLS and their potential as an auxiliary diagnostic tool due to the inflammatory nature of VLS.

## Results

### Characteristics of the participants

Table 1 displays the clinical characteristics of VLS patients. The most common signs or symptoms of VLS were itch. Five out of six patients (83.3%) reported negative effects on sexual function, including libido, arousal, lubrication, orgasm, satisfaction, and pain.

**Table 1:** Characteristics of 6 VLS patients.

	Patient1	Patient 2	Patient3	Patient4	Patient 5	Patient 6
Age	64	58	53	55	66	68
Course	3years	4years	1year	3years	10years	5years
Number of children Born	3	2	1	2	2	2
Affected area	Labia majora	Labia majora	Labia majora, Labia minora	Labia majora, Perieum	Labia majora, labia minora, Perineum, Peirianal	Labia majora
Concomitant symptom	Itch	Itch	Itch	Itch	Itch, pain, Dyspareunia dysuria	Itch, pain
NRS	7	5	7	8	10	6
Skindex-16	36	32	42	64	76	62

### Gut microbial characterization between healthy controls and VLS patients

**Figure 1:**
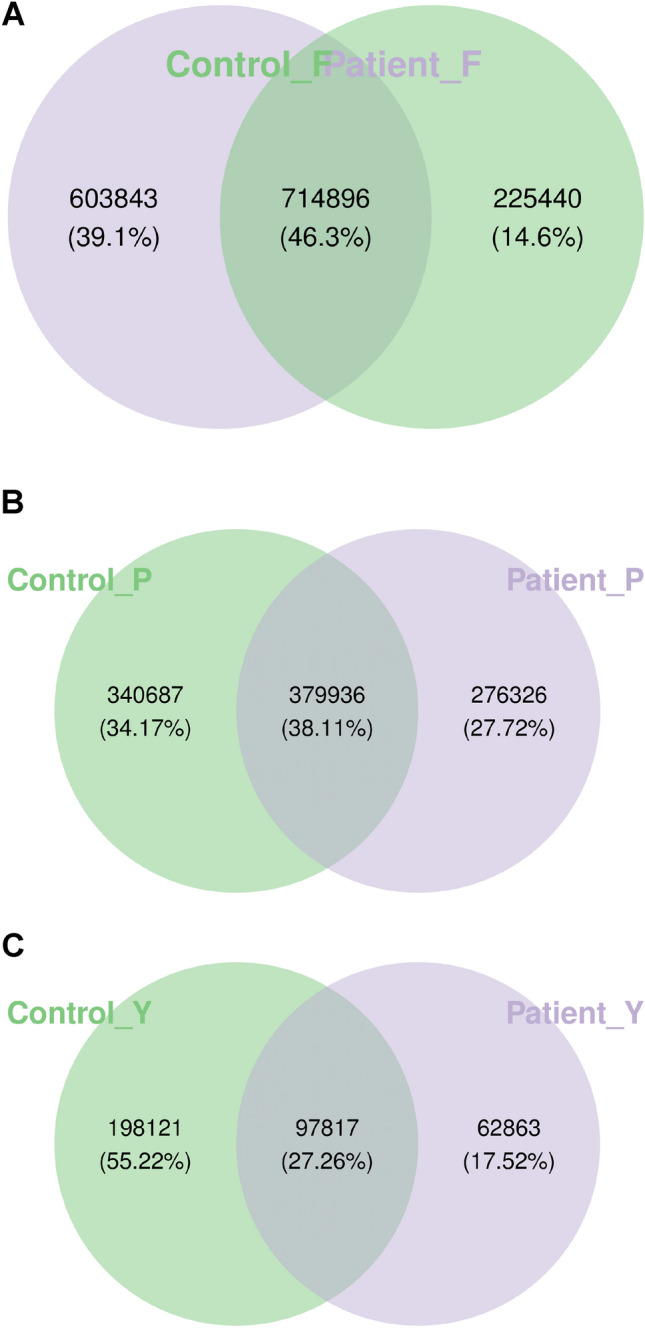
Venn diagram to show gene abundance in patients and controls. Venn diagram of gut group. A total of 844,179 gene abundances were obtained, with 603,843 for the control group and 225,440 for the VLS group. The two groups had 714,896 overlapping genes. (**A**) Venn diagram of skin group. A total of 996,490 gene abundances, with 340,678 for controls and 276,326 for VLS. There were 379,936 overlapping genes between the two groups. (**B**) Venn diagram of vagina group. A total of 358,801gene abundances with 198,121 were from the control group and 62,863 were from the VLS group. The two groups shared 97,817 genes. Con F: represent control group of gut P-F: represent patient group of gut. Con Y: represent control group of vagina P-Y: represent patient group of vagina. Con P: represent control group of skin P-P: represent patient of skin.

**Figure 2:**
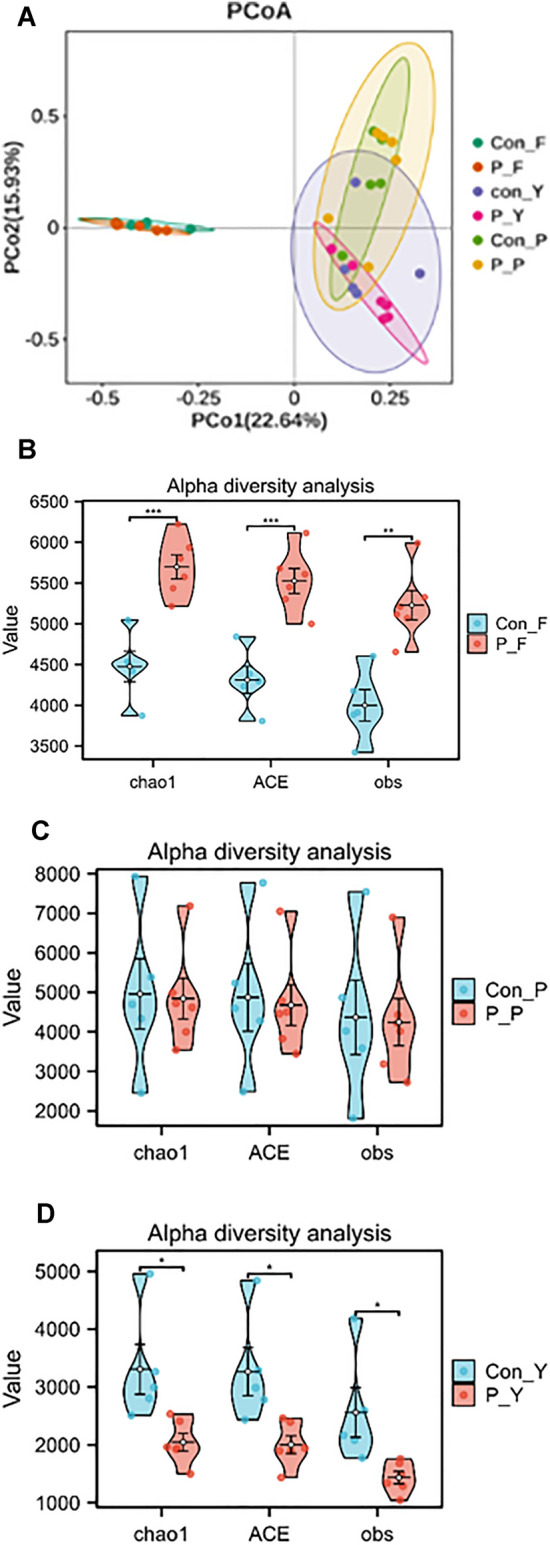
Bacterial characteristics in patients and controls. (**A**) PCoA plots based on bacterial composition at the species level of six groups; (**B**) Changes in the alpha diversity of the ACE, Chao1 index and observed species of GUT groups; (**C**) Changes in the alpha diversity of the ACE, Chao1 index and observed species of SKIN groups; (**D**) Changes in the alpha diversity of the ACE, Chao1 index and observed species of VAGINA groups. Con F: represent control group of gut P-F: represent patient group of gut. Con Y: represent control group of vagina P-Y: represent patient group of vagina. Con P: represent control group of skin P-P: represent patient of skin.

To analyze the distribution of gene numbers and compare the common and unique genes between healthy controls and VLS patients, a Venn diagram was created (Fig. 1A). A total of 844,179 gene abundances were obtained, with 603,843 for the control group and 225,440 for the VLS group. The two groups had 714,896 overlapping genes. Alpha diversity analysis measures species diversity within a biological environment. The ACE Chao 1 and obs richness were significantly higher in the VLS group than in the control group (P < 0.001) (Fig. 2B). Weighted PCOA was used to compare the taxa composition of the two cohorts, and it differentiated between the two groups. However, the Anosim test did not reveal any significant differences at the phylum, species, or genus levels (R < 0, P > 0.05) (Fig. 2A).

**Figure 3:**
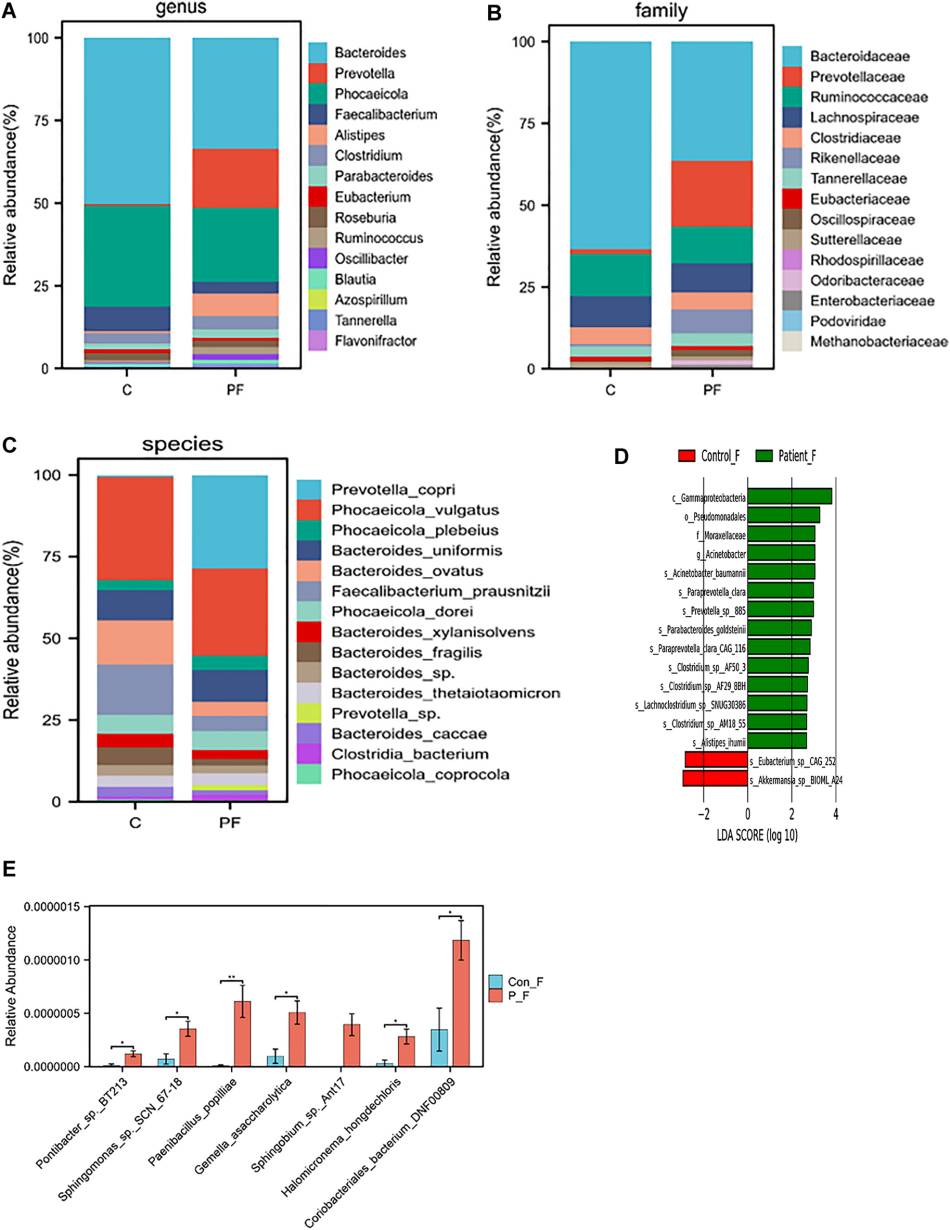
Comparison of gut bacterial community compositions based on bacterial (Operational Taxonomic Unit) between different groups. The relative abundance of top 10 genus (**A**), family (**B**), species (**C**), taxa enriched in patients are indicated with a positive LDA score, and control are indicated with a negative score. Only taxa meeting an LDA significance threshold of > 2 are shown (**D**), and the significantly different at species level (**E**). (*p < 0.05, **p < 0.01, ***p < 0.001, ****p < 0.0001, the p value was adjusted using the Welch t' test method). Con F: represent control group of gut P-F: represent patient group of gut.

In our study, we identified 439 families, 1783 genera, and 10,670 species in gut samples. Figure 3A–C displayed the average composition and relative abundance of the gut microbiome at the species, family, and genus levels. The patient group’s top five dominant bacteria at the genus level were *Bacteroides, Prevotella, Phocaeicola, Faecalibaterium*, and *Alistipes*, accounting for 80% of the microbes. The control group was dominated by *Bacteroides, Phocaeicola, Faecalibacterium, Clostridium, *and *Roseburia*. Upon Analysis at the species level, it was found that the most abundant bacteria in the patient were *Pontibacter_sp._BT213*, *Sphingomonas_sp._SCN_67-18, Paenibacillus_popilliae,Gemella_asaccharolytica,Sphingobium_sp._Ant17,Halomicronema_hongdechloris* and *Coriobacteriales_bacterium_DNF00809*. The group considered healthy showed enrichment with *s-Eubacterium-sp-CAG-252* and *s-Akkermansia-sp-BIOML-A24* (Fig. 3D,E).

Microbial functionality can provide valuable insights into the role of the gut microbiome in host metabolism.The analysis identified a total of 183 pathways with 4 showing statistically differences by T test (P < 0.05). The patient group showed enrichment of homologous recombination, aminoacyl tRNA biosynthesis, toluene degradation, caprolactam degradation, steroid biosynthesis, glutathione metabolism, and sesquiterpenoid and triterpenoid biosynthesis compared to the control group. In contrast, the control group displayed enrichment of the pentose phosphate pathway (Fig. 6A).

### Differences in skin bacteria between healthy volunteer and VLS

The study investigated the bacterial composition and diversity of the skin in two groups. A Venn diagram was used to show the total of 996,490 gene abundances, with 340,678 for controls and 276,326 for VLS. There were 379,936 overlapping genes between the two groups (Fig. 1B). The ACE, Chao1and obs richness indicated that the control group had higher diversity than the VLS patient group, but the difference was not statistical significantly (P > 0.001) (Fig. 2C).We found no significant differences at the phylum, species, or genus level as measured by the Anosim test (R < 0, P > 0.05) (Fig. 2A).

**Figure 4:**
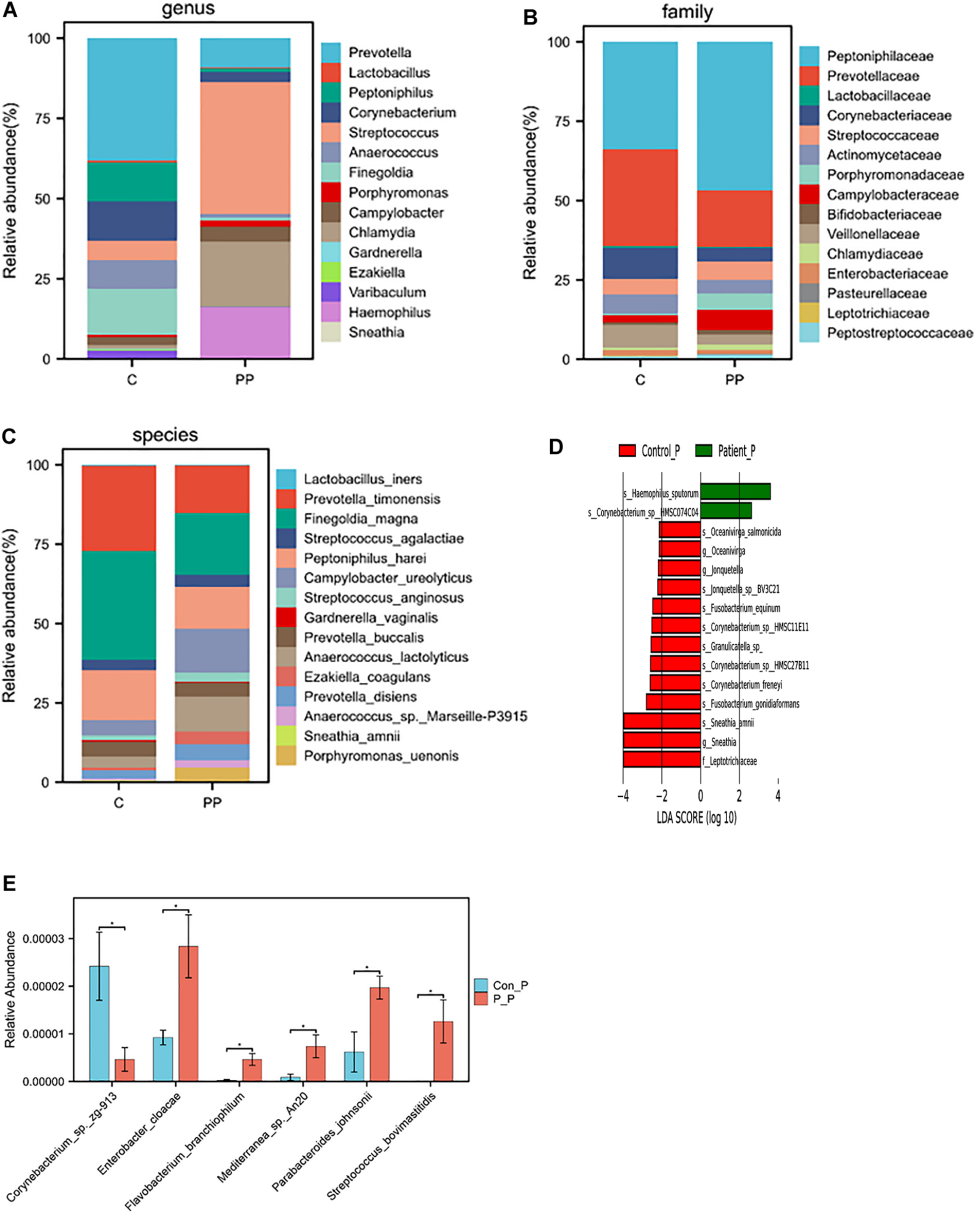
Comparison of skin bacterial community compositions based on bacterial (Operational Taxonomic Unit) between different groups. The relative abundance of top 10 genus (**A**), family (**B**), species (**C**), taxa enriched in patients are indicated with a positive LDA score, and control are indicated with a negative score. Only taxa meeting an LDA significance threshold of > 2 are shown (**D**), and the significantly different at species level (**E**). (*p < 0.05, **p < 0.01, ***p < 0.001, ****p < 0.0001, the p value was adjusted using the Welch t' test method). Con P: represent control group of skin P-P: represent patient of skin.

The study identified 478 families, 1946 genera and 11,408 species. At species level, six showed statistically significant differences. Figure 4A–C display the average composition and relative abundance of the skin microbiome at the genus, family, and species levels. At the genus level, the patient group exhibited dominance of five bacteria: *Streptococcus, Chlamydia, Haemophilus, Prevotella*, and *Campylobacter*. In contrast, the control group exhibited dominance of *Anaerococcus, Peptoniphilus, Corynebacterium, Finegoldia*, and *Prevotella* (Fig. 4D). At the species level, the control group exhibited higher levels of *Corynebacterium_sp._zg-913*, while the patient group had higher levels of *Enterobacter_cloacae, Flavobacterium_branchiophilum, Mediterranea_sp._An20*, *Parabacteroides_johnsonii*, and *Streptococcus_bovimastitidis* (Fig. 4E).

The study compared the functional composition of the skin microbiome in patients with VLS and controls. A total of 230 pathways were identified in the KEGG level 3 pathway analysis. The pathway for alanine, aspartate, and glutamate metabolism was enriched in the patient group (Fig. 6B).

### Differences in vagina bacteria between healthy volunteer and VLS

The study investigated the bacterial composition and diversity of the vagina in two groups. A Venn diagram was used to display the gene abundances, which totaled 358,801. Of these, 198,121 were from the control group and 62,863 were from the VLS group. The two groups shared 97,817 genes (Fig. 1C). Significant differences in α diversity were observed between the two groups, with the ACE Chao 1 and obs richness being higher in the control group than in the patient group(P < 0.005) (Fig. 2D). There were no significant differences at the phylum, species, or genus levels, as measured by the Anosim test (R < 0, P > 0.05) (Fig. 2A).

**Figure 5:**
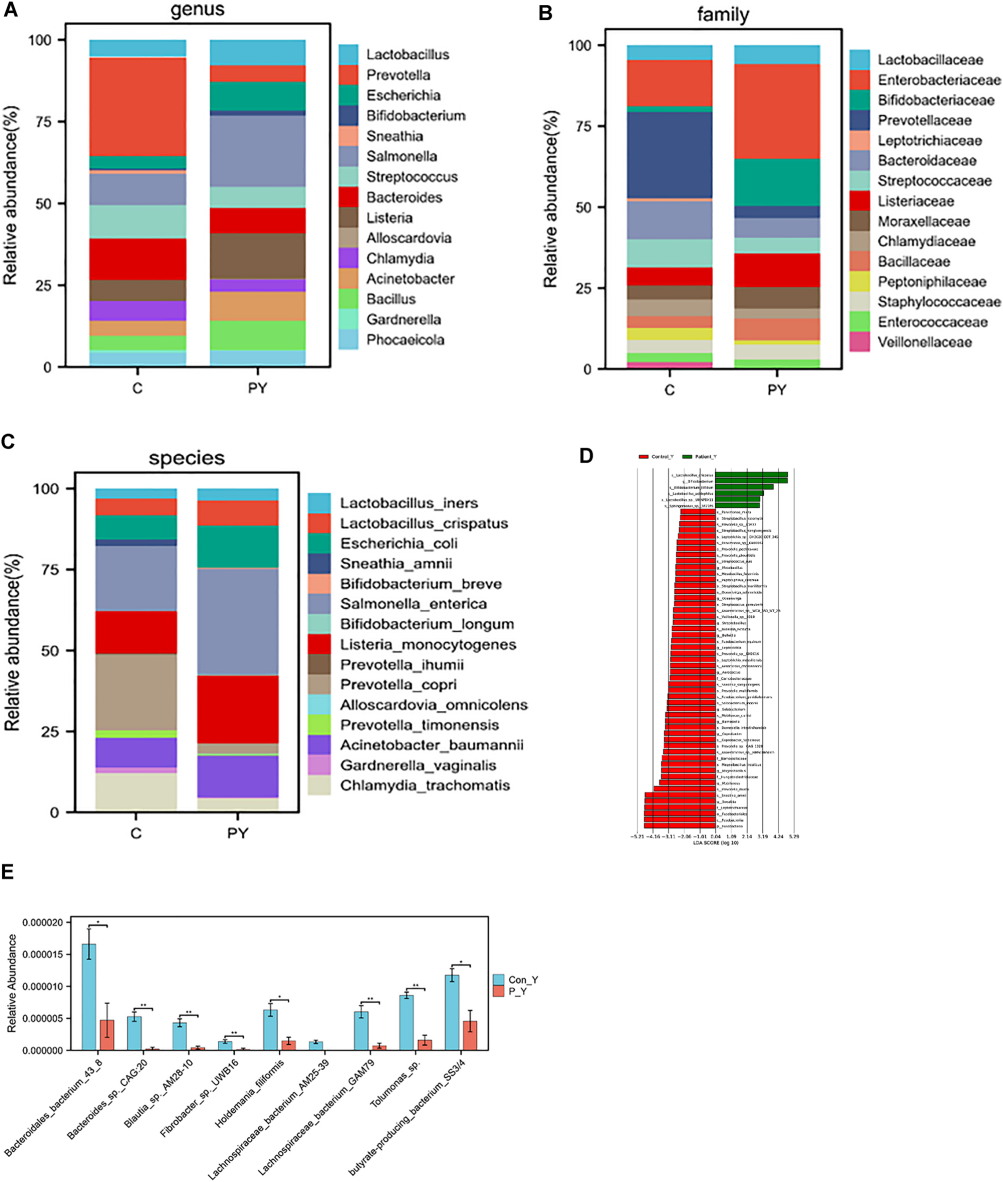
Comparison of vagina bacterial community compositions based on bacterial (Operational Taxonomic Unit) between different groups. The relative abundance of top 10 genus (**A**), family (**B**), species (**C**), taxa enriched in patients are indicated with a positive LDA score, and control are indicated with a negative score. Only taxa meeting an LDA significance threshold of > 2 are shown (**D**), and the significantly different at species level (**E**). (*p < 0.05, **p < 0.01, ***p < 0.001, ****p < 0.0001, the p value was adjusted using the Welch t' test method). Con Y: represent control group of vagina P-Y: represent patient group of vagina.

We identified 303 families, 1068 genera, and 5906 species. Figure 5A–C display the average composition and relative abundance of the skin microbiome at the genus, family, and species levels. The patient group exhibited top five dominant bacteria at the genus level: *Salmonella, Listeria, Bacillus, Acinetobacter,* and *Escherichia*. The control group, on the other hand, had dominant levels of *Listeria, Streptococcus, Salmonella, Bacteroides*, and *Prevotella* (Fig. 5D). Further analysis at the species level showed that the most abundant bacteria in the control group were *Corynebacterium_sp._zg-913, Bacteroidales_bacterium_43_8Bacteroides_sp._CAG:20Blautia_sp._AM28-10Fibrobacter_sp._UWB16,Lachnospiraceae_bacterium_AM25-39, Holdemania_filiformis, Lachnospiraceae_bacterium_GAM79, Tolumonas_sp, butyrate-producing_bacterium_SS3/4* (Fig. 5E).

**Figure 6:**
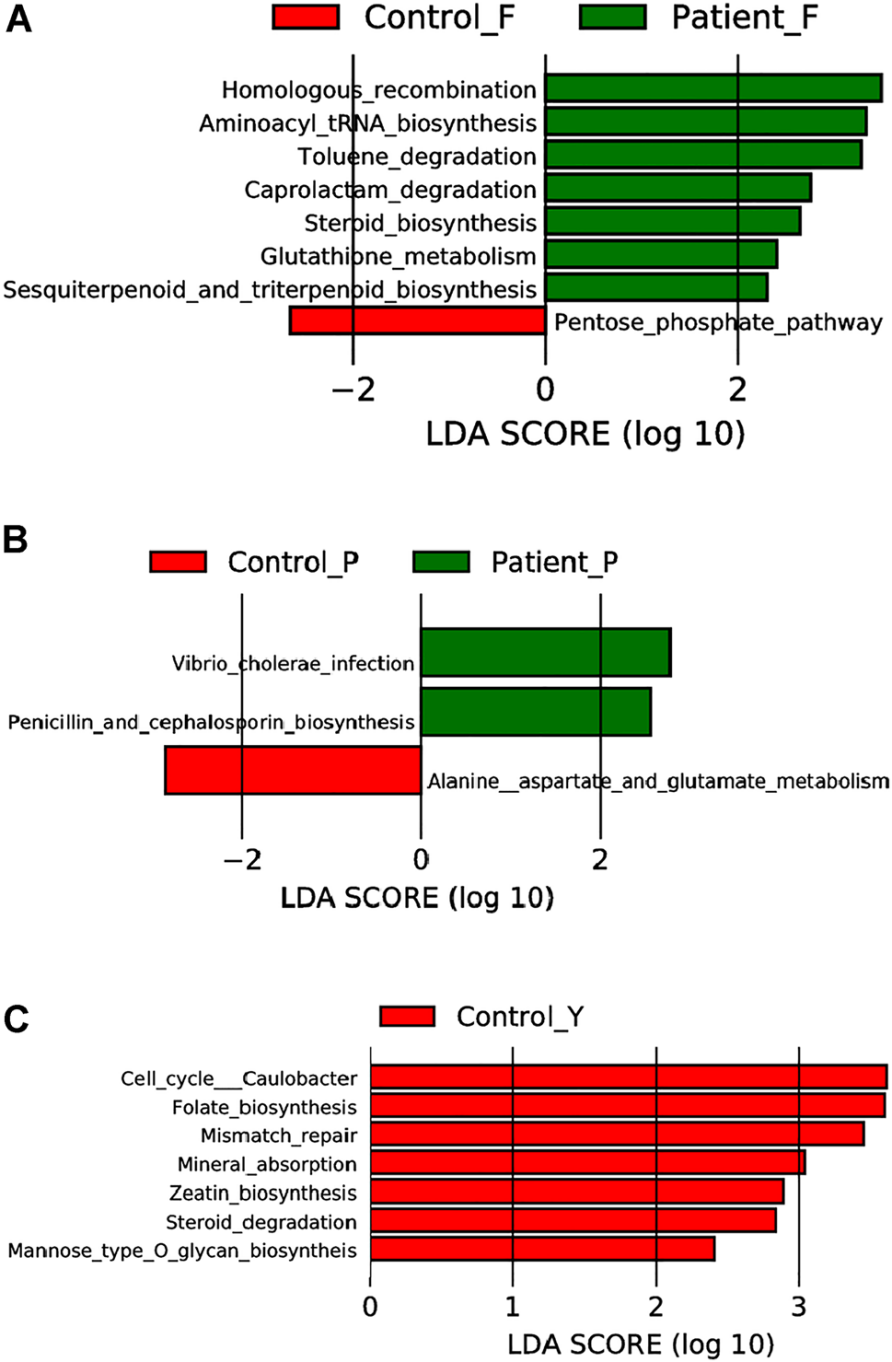
KEGG level 3 abundant characteristics in patients and controls. (**A**) LDA coupled with effect size measurements identifies the most differentially KEGG level 3 abundant taxon's among feces groups. (**B**) LDA coupled with effect size measurements identifies the most differentially KEGG level 3 abundant taxon's among skin groups. (**C**) LDA coupled with effect size measurements identifies the most differentially KEGG level 3 abundant taxon's among vagina groups. Con F: represent control group of gut P-F: represent patient group of gut. Con Y: represent control group of vagina P-Y: represent patient group of vagina. Con P: represent control group of skin P-P: represent patient of skin.

The study compared the functional composition of the fecal microbiome between VLS patients and controls. In the KEGG level 3 pathway analysis, 175 pathways were identified. The patient group showed enrichment of Cell cycle—Caulobacter, Folate biosynthesis, Mineral absorption, Steroid degradation, Mannose type O-glycan biosynthesis (Fig. 6C).

**Figure 7:**
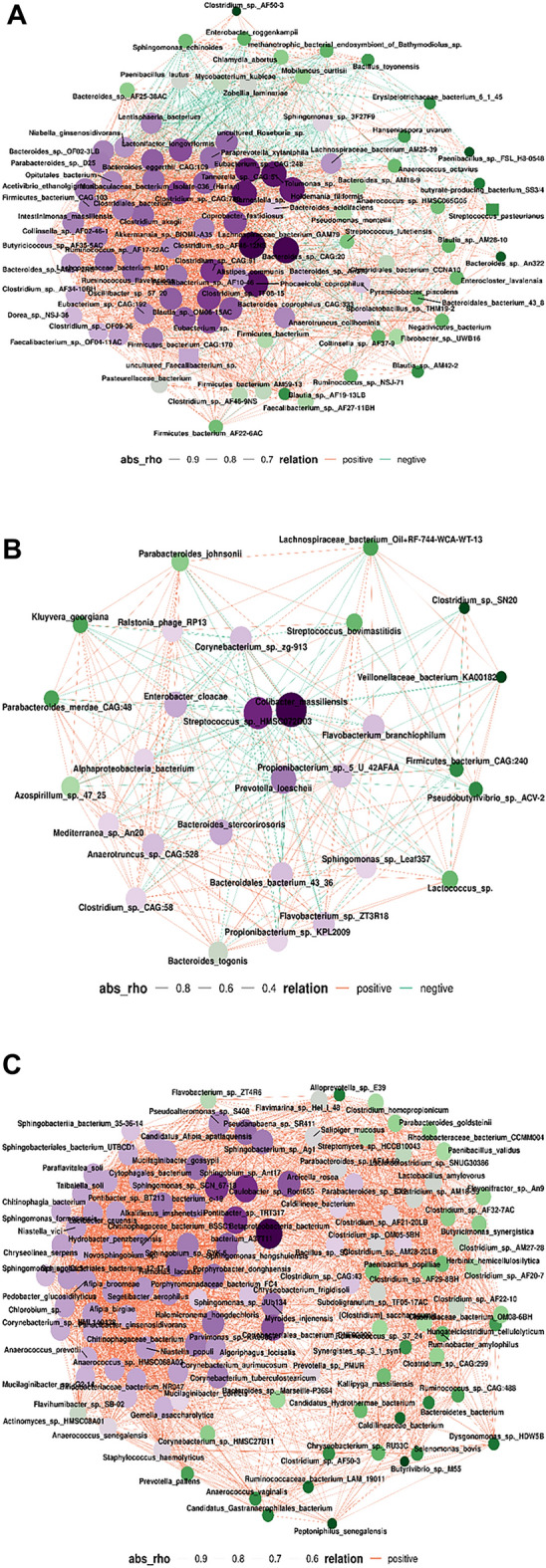
Co-occurrence network deduced from bacterial taxa differentially enriched in the six groups. (**A**), gut (**B**), skin (**C**), vagina. Red edges indicate positive correlations and green edges indicate negative correlations (Spearman, P < 0.05, r > 0.4).

Furthermore, a species co-abundance network was constructed to provide an overview of the interplay among these discriminative bacterial species. As shown in Fig. 7A–C, bacterial species within the same genus clade displayed a significant correlation with each other.

### Linkages between the microbiome and NRS, SKINDEX16

**Figure 8:**
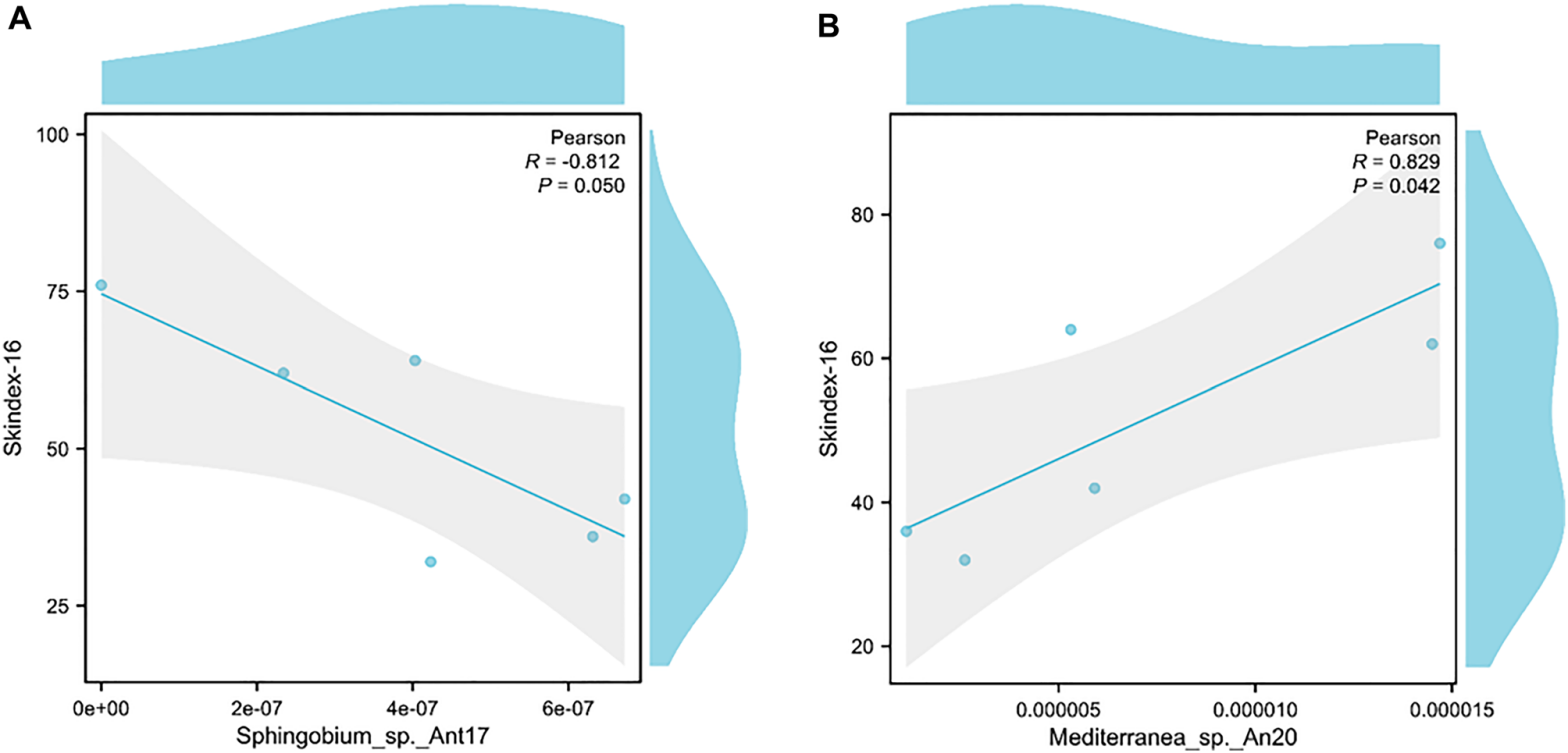
The Pearson correlation analysis was applied to analysis the associations of the differential microbial at the species level with the measured clinical factor. The heat map of correlation coefficient, the red represents positive correlation, and the blue represents negative correlation, respectively (*p < 0.05, **p < 0.01). 8A. In gut; Sphingobium_sp._Ant17 was negatively correlated with Skindex-16. 8B. In skin: Mediterranea_sp._An20 was positively correlated with Skindex-16.

To determine if a specific change in the microbiome is linked to NRS and Skindex-16 in VLS, we analyzed the correlation between the microbiome and these two clinical indicators.The Pearson correlation heatmap revealed that in stool, *Sphingobium_sp._Ant17* had a negative correlation with Skindex-16 (P < 0.05). *Mediterranea_sp._An20* was found to have a positive correlation with Skindex-16 in skin (P < 0.05) (Fig. 8A,B). These results indicate that specific changes in the skin and gut microbiome are strongly linked to clinical indicators.

## Discussion

Skin microbes reside on and interact with the skin, significantly affecting the its barrier function. According to a study conducted by Wolf et al.^18^, using skin grafts demonstrated that a graft placed on the vulva developed LS, and skin with LS from the vulva became normal when grafted to the thigh suggesting that the pathogenesis of VLS may be associated with the local microenvironment. Further analysis was conducted to explore changes in skin microbiota. We revealed that patients with VLS have a higher relative abundance of *Prevotella* at the genus level and *Parabacteroides johnsonii sp.nov* at the species level in the skin. Watchorn^19^ and Chattopahyay^20^ both reported a significantly higher relative abundance of *Prevotella* in male sclerosing lichens and the vulvar flora of VLS children compared to healthy controls. *Prevotella spp*. has been shown to evade the immune system by secreting the bacterial proteases interpain A^21^. These bacterial proteases cleave complement, inhibiting opsonization and phagocytosis, and allowing bacterial overgrowth which increases inflammation^22–24^. Our results are consistent with previous studies, suggesting that *Prevotella* may play a significant role in the pathology of VLS. Additionally, we observed a decrease *Corynebacterium* in the patient group. Previous studies have shown that *Corynebacterium* is the predominant bacteria found on wet skin^25,26^.This finding is consistent with the clinical condition of the dry vulvar skin in patients with vulvar lichen sclerosus.

Additionally, recent studies have reported a close relationship between *Parabacteroides* and host health^27^. For instance, the abundance of *Pa. Distasonis* levels have been found to decrease in various human diseases, including obesity^28^, inflammatory bowel disease^29^, metabolic syndrome^30^, multiple sclerosis^31^, psoriatic disease^32^, and neonatal cholestasis disease^33^. Conversely, distasonis levels have been found to increase in human diseases such as alopecia areata^34^, ankylosing spondylitis^35^, and gestational diabetes mellitus (GDM)^36^. In addition, *Parabacteroides* have been found to possess the physiological characteristics related to carbohydrate metabolism and secretion of short-chain fatty acids. Moreover, the combined use of *Pa. distasonis, Pa. gordonii, Pa. johnsonii*, and eight other bacterial strains can effectively induce interferon-γ-producing CD8 T cells and enhance the anti-tumor immune response^37^. Since an autoimmune phenotype has been observed in cases of VLS, which involves increased levels of Th1-specific cytokines^38^, it is speculated that *Parabacteroides* may play a pathogenic role in VLS through this inflammatory mechanism.

Cumulative evidence has shown that skin and intestine have a bidirectional connection. This study demonstrates that patients with VLS have a distinct gut microbiome signature. In our study, we found an increase in *Roseburia* in the control group, which is consistent with the findings of *Chattopahyay*^20^. *Roseburia spp*. are commensal bacteria that produce short-chain fatty acids, especially butyrate, which affect colonic motility, immune system maintenance, and anti-inflammatory properties^39^. At the species level, *Coriobacteriales, Gemella asaccharolytica*, and *Sphingobium sp. Ant17* showed a significant increase in VLS.

*Coriobacteriales* is an order within the phylum *Actinomycetes* that is associated with several diseases. It has been found to be enriched in tuberculosis (TB)^40^ and type 2 diabetes^41^, but decreased in Type 1 Narcolepsy^42^, Crohn's disease, and ulcerative colitis^43^. These findings suggested that *Coriobacteriaceae* may play a crucial role in maintaining the stability of the intestinal environment. A recent study discovered that *Coriobacteriales* may play a crucial role in the relationship between NO2 exposure and liver function in schizophrenia patients with liver dysfunction^44^. *Coriobacteriaceae* and its genera produce short-chain fatty acids, which are closely linked to host health. Therefore, it is speculated that the disruption of SCFAs may be the key factor linking *Coriobacteriaceae* and VLS.

*Gemella* species are Gram-variable cocci that are facultatively anaerobic and catalase-negative. *Gemella asaccharolytica* was first isolated from a human patient who was an intravenous drug abuser in 2010^45^. A 10-year follow-up study published by McClelland RS found that a higher diversity in vaginal microbiota was associated with an increased risk of HIV infection in a high-risk group of women. The study identified seven microorganisms, including *Gemella asaccharolytica* that were positively associated with the risk of infection^46^. Melis N et al. suggested that antigen-presenting cells sense products of *Gemella asaccharolytica* in situ via Toll-like receptor 4 signaling. This contributes to local inflammation through activation of the NF-κB signaling pathway and recruitment of lymphocytes by chemokine production^47^. This study proposed a mechanism in which the presence of certain bacterial taxa may increase susceptibility to infection by causing inflammation in the vagina.

Similarly, *Sphingobium_sp._Ant17* was found to have a higher relative abundance in the VLS, which is consistent with findings from other inflammatory diseases, such as periodontitis and rheumatoid arthritis in the salivary microbiota. Previous studies have reported a positive relationship between *Sphingobium* and inflammatory mediators, such as TWEAK/TNFSF12, pentraxin-3, and IL-19^48^. In this study, a negative correlation was observed between the change in *Sphingobium sp* and Skindex-16.

The incidence of LS was the highest in women during the period of low estrogen physiological state, which suggested that hormones had an effect on the pathogenesis of the disease. There are few studies on whether vaginal flora is involved in the pathogenesis of VLS. In our study, we found a reduction of *Blautia_sp._AM28-10, butyrate-producing bacteria SS3/4, Holdemania filiformisin* and *Lachnospiraceae_bacterium_GAM79* in the patient group compared to the control group. The patterns of vulval dysbiosis observed in this study have also been implicated in other inflammatory conditions.

It is worth noting that *Blautia* is a genus that belongs to the Trichospirillaceae family. *Blautia* has been studied for its potential to alleviate inflammatory and metabolic diseases. Additionally, it has been found to have antimicrobial activity against specific microorganisms. Studies have shown that *Blautia* levels decrease in older patients and in mucosal samples from patients with colorectal cancer, while increasing in patients with irritable bowel syndrome (IBS)^49–52^. Additionally, *Blautia* showed a negative correlation with visceral fat area, a biomarker for cardiovascular and metabolic disease risk associated with obesity. *Blautia* can prevent colonization by pathogen through the production of bacteriocins. It also possesses anti-inflammatory properties and promotes glucose homeostasis by up-regulating the production of regulatory T cells and short-chain fatty acids (SCFA)^53^.

*The bacterium SS3/4 produces butyrate*, a SCFA that serves as the primary energy source for the colonic epithelium. Moreover, butyrate inhibits the expression of proinflammatory cytokines in the colonic mucosa by preventing NF-κB activation^54^. Nan Qin discovered that the abundance of *butyrate-producing bacterium SS3/4* was higher in the control samples than in the H7N9 patients^55^. Furthermore, butyrate decreases intestinal barrier permeability and improves epithelial barrier integrity. Sodium butyrate has been utilized to treat psoriasis and other hyperproliferative skin diseases by regulating several crucial cellular processes such as differentiation, proliferation and apoptosis^56^.

*Holdemania filiformis*, which belongs to the order *Erysipelotrichales*,has been found to be enriched in alopecia and is considered a potential bacterial biomarker associated with Alopecia universalis^34^. In a recent study of patients with malignant melanoma, researchers at the Harold C. Simmons Cancer Center found that those with specific microbes in their gut, including *Holdemania filiformis*^57^, responded better to cancer immunotherapy.

Metagenomics analyses have identified *Lachnospiracea*e as the primary genus in the human intestine. Members of *Lachnospiraceae* are major producers of short-chain fatty acids (SCFAs). SCFAs have been shown to affect chemotaxis and phagocytosis, induce reactive oxygen species, alter cell proliferation and function, have anti-inflammatory, antitumorigenic, and antimicrobial effects, and affect gut integrity^58^. Metabolic syndrome, obesity, diabetes, and chronic spontaneous urticaria are all inflammatoryy conditions involving the *Lachnospiraceae* family or specific taxa^59–61^. *Lachnospiraceae_bacterium_GAM79* as a member of *Lachnospiraceae*, was also found to have abnormal abundance in myositis and pulmonary arterial hypertension^62,63^. These studies provide additional evidence to suggest a potential causal relationship between vulva dysbiosis and inflammatory conditions in VLS.

The microbiome communicates with the host not only through these microorganisms’ ability to establish biofilms, which leads usually to a chronic inflammation^15^, but also through postbiotics, which are metabolic products such as amino acids, glycometabolites, and biotin metabolites. Our functional KEGG analysis indicates that VLS disease is associated with altered energy metabolism, increased oxidative stress, and abnormal amino acid metabolism.

Specifically, we observed alterations in Aminoacyl-tRNA synthetases (ARSs) in the VLS group. Aminoacyl-tRNA synthetases are enzymes that catalyze the aminoacylation of transfer RNAs during protein synthesis^64^. Although commonly referred to as ‘housekeepers’, recent research has shown that they also function as regulators and signaling molecules in immune cell development. Furthermore, they have been found to play a role in various biological processes related to immune diseases such as Vogt-Koyanagi-Harada and Behcet's disease^65^. These findings suggest that amino acid metabolism may be involved in the pathogenesis of VLS.

Abnormal oxidative stress is associated with numerous diseases. Glutathione is a vital antioxidant with various physiological functions and effects. We also observed altered glutathione metabolism in the VLS. Glutathione can combine with free radicals, inhibit the occurrence of oxidation reactions, reduce cell damage, and protect the body from oxidative stress^66^. Glutathione _metabolism is important in aging and the development of various diseases such as Alzheimer's disease, Parkinson's disease, liver disease, cystic fibrosis, HIV, AIDS, and cancer^67^.

Our experiments revealed an abnormal pathway in glucose metabolism. Specifically, we found dysregulation in the Pentose Phosphate Pathway (PPP), which is a glucose-oxidizing pathway that runs parallel to upper glycolysis to produce ribose 5-phosphate and nicotinamide adenine dinucleotide phosphate. This dysregulation has been linked to a decrease in skin health psoriasis^68^. Additionally, the PPP plays a role in skin fibroblasts and keratinocytes. Kinetic 13C-glucose experiments showed that fibroblasts and keratinocytes increase PPP flux in response to oxidative stress from hydrogen peroxide or UV exposure^69^. The non-oxidative PPP may regulate Treg function to prevent autoimmunity. Silencing PPP genes may affect DNA replication control in human fibroblasts^70^. VLS presents with immune and fibrotic abnormalities. Therefore, we hypothesize that abnormal energy metabolism may be involved in the pathogenesis of VLS.

## Conclusion

In conclusion, this study presented a comprehensive analysis of the gut, skin and vaginal microbiome in perimenopausal or postmenopausal patients with VLS in the Chinese population. The abnormal bacterial community composition and metabolic pathway in the case groups suggested that skin, gut, and vaginal dysbiosis may played a role in the pathogenesis of perimenopausal or postmenopausal VLS. These findings could provide a theoretical basis for clarifying the pathogenesis of VLS and new concepts for its treatment. However, the study has limitations due to its small sample size. Additionally, it was not intended to characterize microeukaryotes such as yeasts, viruses within cutaneous, vaginal, or gut microbiotas.Although so, our encouraging results indicate that further expanding subjects and metabolomics analysis may draw more meaningful conclusions.

## Materials and methods

### Participants

The study received approval from the ethics committee of Peking University International Hospital. And all methods were performed in accordance with the relevant guidelines and regulations. Informed consent to participate in the study was obtained from each patient and healthy person before enrolling in the study. Informed consent was obtained from all participants prior to enrollment, including 6 VLS participants with an average age of 60.67 ± 5.65 and 5 healthy controls with an average age 58.45 ± 6.35 from the Beijing area. All study participants had a confirmed diagnosis of VLS and were able to complete the study. The diagnosis of VLS was based on characteristic clinical manifestations and histologic confirmation. Patients with other vulvar skin lesions, such as eczema, genital condyloma, genital herpes, as well as those with diabetes or receiving treatments that could interfere with the study assessment, were excluded. Participants with diarrhea, bloating, constipation, or other gastrointestinal symptoms within the past 3 months were excluded. Healthy volunteers underwent screening for all types of infectious vaginitis, including bacteria, fungi, and trichomonas, before enrollment. The study ensured that all participants were matched for age demographics.

### Skin, vagina and stool sampling

Participants were instructed to provide a stool sample between 6:00 and 10:00 am, which was immediately stored at − 80 °C.

Prior to taking skin and vagina samples, participants cleaned their skin twice with sterile water. A professional operator then scraped the site of the lesion and vagina with cotton swabs. The swab was gently rotated for 30 s on each side, then immediately placed into a cryotube and transferred to the freezer at -80℃for further analysis.

### Itch intensity scales were used to assess the pruritus score

Pruritus severity was evaluated using the Numeric Rating Scale (NRS), which rates itch severity on a ten-point scale, with 0 indicating 'no itch' and 10 indicating 'worst itch imaginable.'

The study used the Skindex-16 scales to evaluate the impact of skin issues on patients’ quality of life (QOL).

Specifically, the Skindex-16 assessed the dermatology-specific health-related quality of life (HrQoL) measures of each patient. The tool includes domain scores that evaluate how symptoms, emotions, and functioning related to the skin issue affect the QOL of patients with VLS. The overall score is an average of the three domain scores, each of which is normalized to a scale of 0 to 100. A score of 0 indicates that the individual’s skin condition has no impact on their quality of life, while a score of 100 represents the maximum negative impact on quality of life.

### DNA isolation and library construction

Total DNA was isolated from sample using a QIAamp^®^ Fast DNA Stool Mini Kit (Qiagen, Hilden, Germany) following the manufacturer’s instructions. DNA concentration and integrity were assessed by a NanoDrop2000 spectrophotometer (Thermo Fisher Scientific, Waltham, MA, USA) and agarose gel electrophoresis, respectively. DNA was fragmented by S220 Focused-ultrasonicators (Covaris, USA) and cleaned up by Agencourt AMPure XP beads (Beckman Coulter Co., USA). Then the libraries were constructed using TruSeq Nano DNA LT Sample Prepararion Kit (Illumina, San Diego, CA, USA) according to the manufacturer’s instructions.

### Bioinformatic analysis

The libraries were sequenced on an llumina Novaseq 6000 platform and 150 bp paired-end reads were generated. Sequences in the FastQ file were trimmed and filtered using Trimmomatic (v 0.36)^71^. Host pollution control was needed. The post-filtered pair-end reads were aligned against the host genome using bowtie2 (v 2.2.9) and the aligned reads were discarded. Metagenome assembly was performed using MEGAHIT (v 1.1.2)^72,73^ after getting valid reads. Use gaps inside scaffold as breakpoint to interrupt the scaffold into new contigs (Scaftig), and these new Scaftigs with length > 500 bp were retained. ORF prediction of assembled scaffolds using prodigal (v 2.6.3) was performed and translated into amino acid sequences. The non-redundant gene sets were built for all predicted genes using CDHIT (v 4.5.7). The clustering parameters were 95% identity and 90% coverage. The longest gene was selected as representative sequence of each gene set. Clean reads of each sample were aligned against the non-redundant gene set (95% identity) use bowtie2 (v 2.2.9), and the abundant information of the gene in the corresponding sample was counted.

The taxonomy of the species was obtained as a result of the corresponding taxonomy database of the NR Library, and the abundance of the species was calculated using the corresponding abundance of the genes. In order to construct the abundance profile on the corresponding taxonomy level, abundance statistics were performed at each level of Domain, Kingdom, Phylum, Class, Order, Family, Genus and Species. The gene set representative sequence (amino acid sequence) was annotated with NR, KEGG^74–76^, eggNOG^77^, SWISSPROT and GO database with an e-value of 1e-5 using DIAMOND (v 0.9.7)^78^. The gene sets were compared with the CAZy database^79^ using the corresponding tool hmmscan (v 3.1) to obtain the information of the carbohydrate active enzyme corresponding to the gene and then calculated the carbohydrate activity using the sum of the gene abundances corresponding to the carbohydrate active enzyme abundance.

The PCA analysis and plotting of the taxonomy abundance spectrum or functional abundance spectrum were carried out using R software (v 3.2.0), and the results of the equidistant matrix of PCoA and NMDS were calculated and analyzed. Then the R package was used to analyze the significant differences between different groups using Wilcoxon statistical test. The linear discriminant analysis effect size (LEfSe) method was used to compare the taxonomy abundance spectrum or functional abundance spectrum.

## Data Availability

The raw data is available from the corresponding author (superma.xiaolei@163.com) on reasonable request.

## Abbreviations

VLS: Vulvar lichen sclerosus
PPP: Pentose Phosphate Pathway
